# The role of sarcopenia in treatment-related outcomes in patients with renal cell carcinoma: A systematic review and meta-analysis

**DOI:** 10.1097/MD.0000000000031332

**Published:** 2022-10-28

**Authors:** Li Yuxuan, Li Junchao, Liu Wenya

**Affiliations:** a Imaging Department, the First Affiliated Hospital of Guangzhou University of Traditional Chinese Medicine, Guangdong, China.

**Keywords:** prognosis, renal cell carcinoma, sarcopenia

## Abstract

in recent years, more attention has been paid to the fuzzy relationship between skeletal muscle components and renal cell carcinoma (RCC). This study attempts to conduct a meta-analysis using all relevant research evidence to explore the impact of sarcopenia on the final survival and recurrence outcome of RCC patients and the change process of this impact after treatment.

**Methods:**

This systematic review and Meta-analysis study took “sarcopenia”, “kidney” and “tumor” and their synonyms as the main search terms, and comprehensively searched all relevant literatures published in PubMed, web of science, SpringerLink, EMBASE, Cochrane Library, Ovid (Lww oup), Wiley, ScienceDirect and Scopus databases since February 2, 2022. Multivariate hazard ratio (HR) and 95% confidence interval (CI) of overall survival (OS), cancer specific survival (CSS), and progression free survival (PFS), as well as rough data of Kaplan–Meier survival curve, were combined as the main analysis results. Subgroup analyses based on cohort characteristics (treatment, ethnicity, and BMI factors) for each study were used as secondary outcomes. The combined effect was estimated by random effect model or fixed effect model, and the heterogeneity was evaluated by *I*^2^ value. Because this study belongs to secondary literature, the medical ethics committee of the First Affiliated Hospital of Xinjiang Medical University considers that ethical review is unnecessary.

**Results:**

Eighteen retrospective studies involving 3591 patients with RCC were analyzed, of which 71.5% were men and the median age of the cohort was 61.6. The prevalence of sarcopenia was 43% (38–48%). Sarcopenia is an independent predictor of OS (HR: 1.83, 95% CI = [1.41, 2.37]), and this prognostic value can also be reflected in Asian populations (HR: 2.59, 95% CI = [1.90, 3.54]) and drug treated patients (HR: 2.07, 95% CI = [1.07, 4.04]). Sarcopenia can also be used as an independent predictor of CSS (HR: 1.78, 95% CI = [1.34, 2.36]) and PFS (HR: 1.98, 95% CI = [1.34, 2.92]). The effect of low skeletal muscle mass on OS and CSS increased slowly from 1 to 5 years.

**Conclusion:**

Sarcopenia can be used as a comprehensive prognostic factor in RCC population, but the detailed effects from ethnic characteristics and treatment mechanism need to be further studied.

## 1. Introduction

According to the study, the incidence rate of kidney cancer has been rising continuously from 2000 to 2020. The number of new cases of kidney cancer increased from 19,000 to about 4,30,000, and the number of new deaths increased from 90,000 to 1,80,000, accounting for about 2% of the new malignant tumors and new deaths in 2020.^[1,2]^ Renal cell carcinoma (RCC) is the most common renal malignancy in modern medicine, and patients with distant metastasis often have poor long-term prognosis. In addition, about one third of patients have distant metastasis at the initial examination, and the remaining 70% are mainly confined to the kidney, which may be due to the popularity of large imaging devices such as CT (CT) and MR (MR), so that tumors can be detected early.^[3]^ Although significant progress has been made in laparoscopic surgery or nephrectomy for RCC compared with the past, which can preserve renal function more completely and minimize cancer progress, However, studies have shown that up to 30% of patients will relapse after tumor resection.^[4]^

At present, tumor stage and Fuhrman’s pathological nuclear grading system are considered to be the most important prognostic factors of RCC, but even so, the improvement of prognosis can only focus on the prolongation of 5-year survival rate rather than the cure of tumor. Therefore, the correct identification of the recurrence and prognostic factors of RCC has always been the core issue of improving the survival time, which is very important for prolonging the survival time, dynamically monitoring the prognostic changes of patients, especially the prediction of cancer recurrence. Recent evidence shows that sarcopenia, characterized by skeletal muscle atrophy, performs well in terms of treatment prognosis, disease recurrence and progression, and survival results. This disease with imbalance of body composition has a variety of pathogenesis, such as forced braking, dysphagia, sympathetic overactivation and muscle denervation, especially closely related to the high metabolic state and high inflammatory level state mediated by some cancers.^[5]^ Although the pathological mechanism of this secondary sarcopenia is still unclear, experiments have found that it will not only become a part of the disease in the course of its onset, and jointly affect the survival and prognosis of patients, but also accelerate the rate of skeletal muscle loss while worsening the clinical results, and fall into a vicious circle of continuous loss of body components. At this stage, myopenia has a special value in predicting the survival prognosis of patients and reflecting the physical state. Different from the clear conclusions in hepatocellular carcinoma,^[6]^ the association in RCC cohort is not so clear. Psutka^[7]^ and Peyton^[8]^ respectively stated that sarcopenia had no effect on the patient’s risk of tumor progression and death. However, Ishihara^[9]^ has come to a different conclusion that sarcopenia defined by L3 skeletal muscle index (SMI) needs to be a very important independent predictor of RCC death and tumor recurrence. Therefore, this study attempts to comprehensively analyze the final impact of sarcopenia on the outcome of death and tumor progression in patients with RCC, and the change process of this impact during follow-up.

## 2. Materials and Methods

### 2.1. Search strategy

This study were performed according to the Preferred Reporting Items for Systematic Reviews and Meta‐Analyses guidelines (CRD 42021287506).^[10]^ We used the following search formula: (muscular Atrophy OR sarcopenia OR sarcopenic OR skeletal muscle depletion OR muscle index OR muscle mass) AND (renal OR kidney OR nephro OR nephritic) AND (cancer OR carcinoma OR tumor OR neoplasm OR cancerous OR carcinomatous) to systematically search the PubMed, Web of science, Springerlink, Embase, cochrane library, Ovid (lww-oup), Wiley, sciencedirect, Scopus database, including any possible relevant articles that have studied the potential relationship between sarcopenia and renal cancer since the first article, as well as the important references cited by them, and there are no language restrictions.

### 2.2. Inclusion and exclusion criteria

Based on all the obtained literature, we used the following inclusion and exclusion criteria for more detailed qualified literature screening: the study cohort clearly defined tumor types and sarcopenia; diagnosis of skeletal muscle loss in any way, including computed tomography, magnetic resonance, dual energy X-ray absorptiometry (DEXA) or bioelectrical impedance analysis (BIA), and functional definitions; and results: multivariate Cox analysis with overall survival (OS), cancer specific survival (CSS) and progression free survival (PFS) as the final outcome, or Kaplan–Meier survival analysis curve with long-term follow-up results. Exclusion criteria: study design/type: meeting abstract, case report, conference report, expert opinion, review, animal or chemical experiment research; tumor type: patients in the cohort had malignant tumors not from the kidney; and data: insufficient survival analysis data or studies were improperly grouped and defined.

### 2.3. Data extraction and quality assessment

Two authors independently collected the following data using the same excel form: first author, year of publication, type of study, country, metastatic situation, treatment mode, sample size, median age, imaging indicators (range and level of skeletal muscle measurement, diagnostic cutoff value) and prognostic indicators (OS, CSS, PFS) and their HR and 95% CI. And the long-term impact process of OS, CSS, and PFS during the follow-up period is dynamically analyzed through the survival data roughly estimated by Kaplan–Meier curve, which will be the main result of this meta-analysis. Since the included literatures are observational studies, we use the Newcastle Ottawa Scale (NOS) to assess the quality of the research,^[11]^ NOS is a validated tool to assess the quality of reporting, as well as the methodological quality of internal (bias and confusion) and external validity. The quality of each study was assessed from three main aspects: selection; the comparability of cohort; and the adequacy of outcome evaluation, duration and follow-up, we defined 7 to 9 as high-Quality study. Before and after applying all the data to get the results, we will review the input data again. If the data collected by the two authors are inconsistent, meta-analysis will be conducted after discussion and consensus.

### 2.4. Statistical analysis

Our research is based on the data from the original article and used STATA14.0 software (StataCorp, College Station, TX) to conduct this meta-analysis. According to the forest map, we use *I*^2^ to evaluate the heterogeneity between the studies. If the analysis result between each group of studies is *P* < .1 or *I*^2^ > 50%, then the random effects model will be used. Otherwise, we used the fixed effect model. In order to explore the impact of heterogeneity in the literature, we delete each study in turn and collect the remaining studies for sensitivity analysis. Secondly, we use the Begg method to make a funnel chart to observe the publication bias. Two-sided *P* < .05 was defined as statistically significant.

## 3. Result

Two independent researchers reviewed the titles and abstracts in each database according to the defined retrieval formula, and 7613 documents that initially met the requirements were obtained. After the list of included studies was confirmed through joint discussion and full-text resources were obtained, 18 literatures were explained in detail and the research process was displayed in detail (Table 1). We extracted 3591 RCC patients who had clearly reported the diagnostic evidence of sarcopenia and completed follow-up, including 1445 sarcopenia and 2146 non-sarcopenia patients, who received surgery, targeted drugs or systematic treatment respectively. According to the data obtained, the median age of the whole study cohort was 61.6 years, of which 71.5% were men. Interestingly, although we did not exclude the definition of sarcopenia from functional aspects when searching the research, the qualified literature retrieved were all the measurements of skeletal muscle quality. Therefore, in this study, we defined low skeletal muscle quality as sarcopenia. The prevalence of sarcopenia in RCC population was 43% (38–48%). Half of these studies were from Eastern countries, including China, Japan and South Korea, and the participants in the other nine studies were from the United States, France, the Netherlands and the Czech Republic (Table 2).

**Table 1:** The characteristics of each study included in the meta-analysis.

First author^[Ref]^, year	Research type	Country	Metastasis	Treatment	Sample size	Median age	Diagnostic index	Cutoff value	OS	CSS	PFS	Score
Noguchi,^[12]^ 2020	Retros	Japan	Localized	Surgery	316	63	L4-PMI	Median	2.167 (1.056, 4.444)	–	–	8
Cushen,^[13]^ 2014	Retros	Netherlands	Metastatic	Chemotherapy	65	66	L3-SMI	Quartile	–	–	–	6
Psutka,^[7]^ 2015	R etros	America	Localized	Surgery	387	–	L3-SMI	Male < 55 cm^2^/m^2^, female < 39 cm^2^/m^2^	1.70 (1.01, 2.85)	1.48 (1.02, 2.15)	1.10 (0.74, 1.63)	7
Peyton,^[8]^ 2015	Retros	America	Metastatic	Surgery	137	66	L3-TPA	Quartile	1.77 (0.88, 4.04)	–	–	8
Gu,^[14]^ 2017	Retros	China	Localized	Multiple	101	–	L3-△SMI	△SMI < 5%	2.367 (1.253, 4.469)	–	–	9
Ishihara*,^[9]^ 2018	Retros	Japan	Localized	Chemotherapy	69	–	L3-△SMI	△SMI < 0	4.53 (2.15, 10.5)	–	3.25 (1.74, 6.29)	9
Darbas,^[15]^ 2020	Retros	France	Localized	Surgery	93	–	L3-SMI	Male < 53 cm^2^/m^2^. female < 41 cm^2^/m^2^	0.5 (0.2, 1.4)	–	–	8
Auclin,^[16]^ 2017	Retros	France	Metastatic	Chemotherapy	124	–	L3-SMI	Male < 55.4 cm^2^/m^2^, female < 38.9 cm^2^/m^2^	1.10 (0.79, 1.54)	–	–	7
Lee*,^[17]^ 2021	Retros	Korea	Metastatic	Chemotherapy	78	61.6	L3-SMI	Male: BMI < 25, value < 55.4cm^2^/m^2^; BMI ≥ 25, value < 53 cm^2^/m^2^. female < 41 cm^2^/m^2^	2.12 (1.17, 3.83)	–	2.63 (1.50, 4.61)	9
Fukushima,^[18]^ 2015	Retro	Japan	Metastatic	Multiple	92	–	L3-SMI	Male: BMI < 25, value < 55.4 cm^2^/m^2^; BMI ≥ 25, value < 53 cm^2^/m^2^. female < 41 cm^2^/m^2^	2.58 (1.20, 6.05)	–	–	9
Higgins*,^[19]^ 2021	Retro	America	Localized	Surgery	352	62.7	L3-SMI	BMI < 30: female < 38 cm^2^/m^2^; male < 47 cm^2^/m^2^, BMI ≥ 30: female < 47 cm^2^/m^2^; male < 54 cm^2^/m^2^	1.64 (1.15, 2.34)	2.01 (1.19, 3.39)	–	7
Ishihara †,^[20]^ 2016	Retro	Japan	Localized	Chemotherapy	71	64	L3-SMI	Male: BMI < 25, value < 55.4 cm^2^/m^2^; BMI ≥ 25, value < 53 cm^2^/m^2^. female < 41 cm^2^/m^2^	2.29 (0.73, 8.16)	–	2.54 (1.19, 5.65)	8
Mao*,^[21]^ 2020	Retro	China	Metastatic	Surgery	336	58.02	L3-SMI/TPI	SMI—male:BMI < 25, value < 55.4 cm^2^/m^2^; BMI ≥ 25, value < 53 cm^2^/m^2^. female < 41 cm^2^/m^2^ TPI—male < 54.5 cm^2^/m^2^，female < 38.5 cm^2^/m^2^	–	–	–	6
Sharma,^[22]^ 2015	Retro	USA	Metastatic	Multiple	93	61	L3-SMI	Male: BMI < 25, value < 55.4 cm^2^/m^2^; BMI ≥ 25, value < 53 cm^2^/m^2^. female < 41 cm^2^/m^2^	2.127 (1.153, 3.924)	–	–	9
Buchler,^[23]^ 2020	Retro	Czech	Metastatic	Multiple	54	65.5	L3-SMI	Male < 55 cm^2^/m^2^，female < 39 cm^2^/m^2^	–	–	1.712 (0.297, 9.863)	7
Lee†,^[24]^ 2021	Retro	Korea	Localized	Surgery	632	54	L3-SMI	Male < 52.4 cm^2^/m^2^，female < 38.5 cm^2^/m^2^	2.58 (1.02, 6.54)	3.07 (1.38, 6.83)	–	8
Mao§,^[25]^ 2021	Retro	China	Metastatic	Surgery	343	57.47	L3-SMI	Male:BMI < 25, value < 55.4 cm^2^/m^2^; BMI ≥ 25, value < 53 cm^2^/m^2^. female < 41 cm^2^/m^2^	–	–	–	7
Huillard,^[26]^ 2013	Retro	France	Metastatic	Chemotherapy	61	60	L3-SMI	Male < 55.4 cm^2^/m^2^, female < 38.9 cm^2^/m^2^	–	–	–	6

BMI = body mass index, CSS = cancer specific survival, MI = change rate of skeletal muscle index, OS = overall survival, PFS = progression free survival, PMI = psoas muscle index, SMI = skeletal muscle index, TPI = total psoas index.

†,‡ Represent different studies published by the same author at different times.

**Table 2:** Main outcomes extracted from the studies included in our meta-analysis.

First author,^[Ref]^ year	OS				CSS				PFS			
	Sarcopenia		Non-sarcopenia		Sarcopenia		Non-sarcopenia		Sarcopenia		Non-sarcopenia	
	1-yr	5-yr	1-yr	5-yr	1-yr	5-yr	1-yr	5-yr	1-yr	5-yr	1-yr	5-yr
Noguchi,^[12]^ 2020	72.3%	15.4%	80.2%	18.5%	–	–	–	–	–	–	–	–
Cushen,^[13]^ 2014	–	–	–	–	–	–	–	–	68.0%	7.0%	92.5%	16.5%
Psutka,^[7]^ 2015	88.0%	64.0%	96.0%	74.0%	93.5%	78.0%	97.0%	85.0%	93.0%	65.0%	81.0%	68.5%
Peyton,^[8]^ 2015	93.7%	51.0%	87.0%	48.0%	–	–	–	–	–	–	–	–
Gu,^[14]^ 2017	37.5%	7%	62%	25%	–	–	–	–	–	–	–	–
Ishihara*,^[9]^ 2018	77.5%	17%	87.5%	43.5%	–	–	–	–	25.5%	5.0%	74.0%	18.0%
Darbas,^[15]^ 2020	94.0%	65.0%	93.5%	80.5%	–	–	–	–	–	–	–	–
Auclin,^[15]^ 2017	–	–	–	–	–	–	–	–	–	–	–	–
Lee*,^[17]^ 2021	75.7%	5%	43.9%	2%	–	–	–	–	22.0%	0.0%	56.8%	0.0%
Fukushima,^[18]^ 2015	74.0%	16.0%	92.5%	62.5%	91.5%	64%	97.5%	94.5%	–	–	–	–
Higgins*,^[19]^ 2021	86.8%	52.0%	96.0%	73.5%	–	–	–	–	–	–	–	–
Ishihara†,^[20]^ 2016	65.0%	35.0%	95.0%	56.0%	–	–	–	–	26.0%	0.0%	71.5%	20.5%
Mao*,^[21]^ 2020	90.5%	69.0%	96.5%	90.0%	94.0%	79.2%	98.0%	93.0%	–	–	–	–
Mao*,^[21]^ 2020	86.0%	69.0%	96.8%	87.0%	93.0%	80.5%	98.5%	89.0%	–	–	–	–
Sharma,^[22]^ 2015	36.0%	3.5%	64.0%	32.0%	–	–	–	–	–	–	–	–
Buchler,^[23]^ 2020	–	–	–	–	–	–	–	–	43.5%	–	60.5%	–
Lee†,^[24]^ 2021	95.0%	81.0%	100%	93.5%	96.5%	88.0%	100%	99.0%	–	–	–	–
Mao‡,^[25]^ 2021	93.5%	77.5%	96.0%	94.0%	95.0%	76.0%	96.0%	92.5%	–	–	–	–
Huillard,^[26]^ 2013	72.5%	–	67.5%	–	–	–	–	–	32.0%	–	46.8%	–

CSS = cancer specific survival, OS = overall survival, PFS = progression free survival.

†, ‡, § Represent different studies published by the same author at different times.

### 3.1. Effects of sarcopenia on OS, CSS, and PFS outcomes from different levels

**Figure 1.**
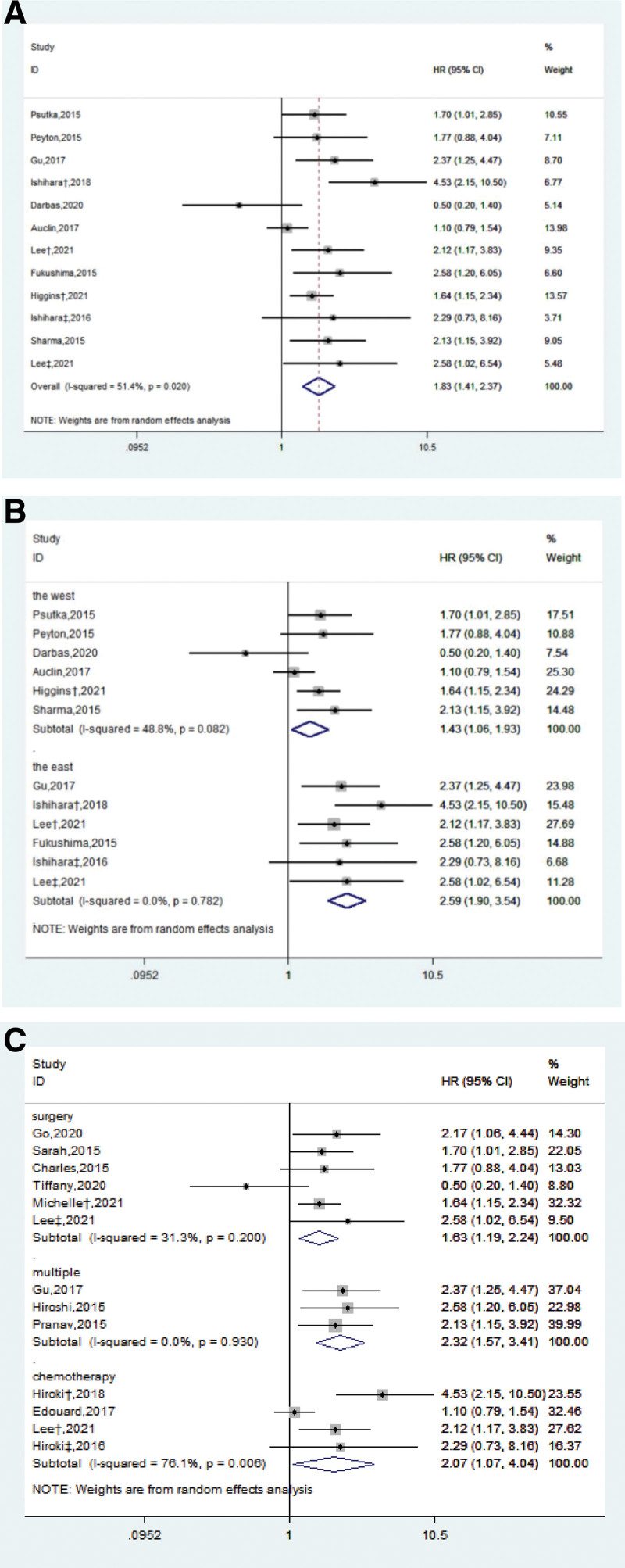
Forest map of overall survival, as well as subgroup analysis of race and treatment. ^†,‡^ Represent different studies published by the same author at different times. CI = confidence interval, HR = hazard ratio.

We extracted the results of multivariate analysis of sarcopenia from all included studies, of which 13 studies reported the OS results of Cox in 2545 patients with RCC. After integrating the data, it was found that sarcopenia was an independent predictor of the risk of death in patients with RCC (HR: 1.83, 95% CI = [1.41, 2.37], *I*^2^ = 51.4%) (Fig. 1A). After racial division of these study participants, we found that RCC patients with sarcopenia in Asia (HR: 2.59, 95% CI = [1.90, 3.54], *I*^2^ = 0%) faced almost twice the risk of death as the western population (HR: 1.43, 95% CI = [1.06, 1.93], *I*^2^ = 48.8%) (Fig. 1B). Meanwhile, in the process of literature feature extraction, we noticed that many literatures reported the treatment methods of patients in detail. Subgroup analysis showed that sarcopenia in patients with renal cancer had prognostic value in routine surgical treatment,including partial nephrectomy and radical nephrectomy (HR: 1.63, 95% CI = [1.19, 2.24], *I*^2^ = 31.3%), but the risk of death from skeletal muscle loss caused by drug treatment seemed to be greater (HR: 2.07, 95% CI = [1.07, 4.04], *I*^2^ = 76.1%) (Fig. 1C).

Three^[7,19,24]^ of these studies reported a specific risk of cancer-mediated death in 1371 patients. Meta-analysis showed that sarcopenia still maintained a similar prognostic value (HR: 1.78, 95% CI = [1.34, 2.36], *I*^2^ = 31.8%) (Figure S1, Supplemental Digital Content, http://links.lww.com/MD/H732). Ishihara^[9]^ and other five authors studied the relationship between body composition and cancer progression and made similar findings (HR: 1.98, 95% CI = [1.34, 2.92], *I*^2^ = 57.4%), which can also be extended to Asian populations (HR: 2.34, 95% CI = [1.75, 3.15], *I*^2^ = 0%) (Figure S2, Supplemental Digital Content, http://links.lww.com/MD/H733).

### 3.2. Sarcopenia measurement

**Figure 2.**
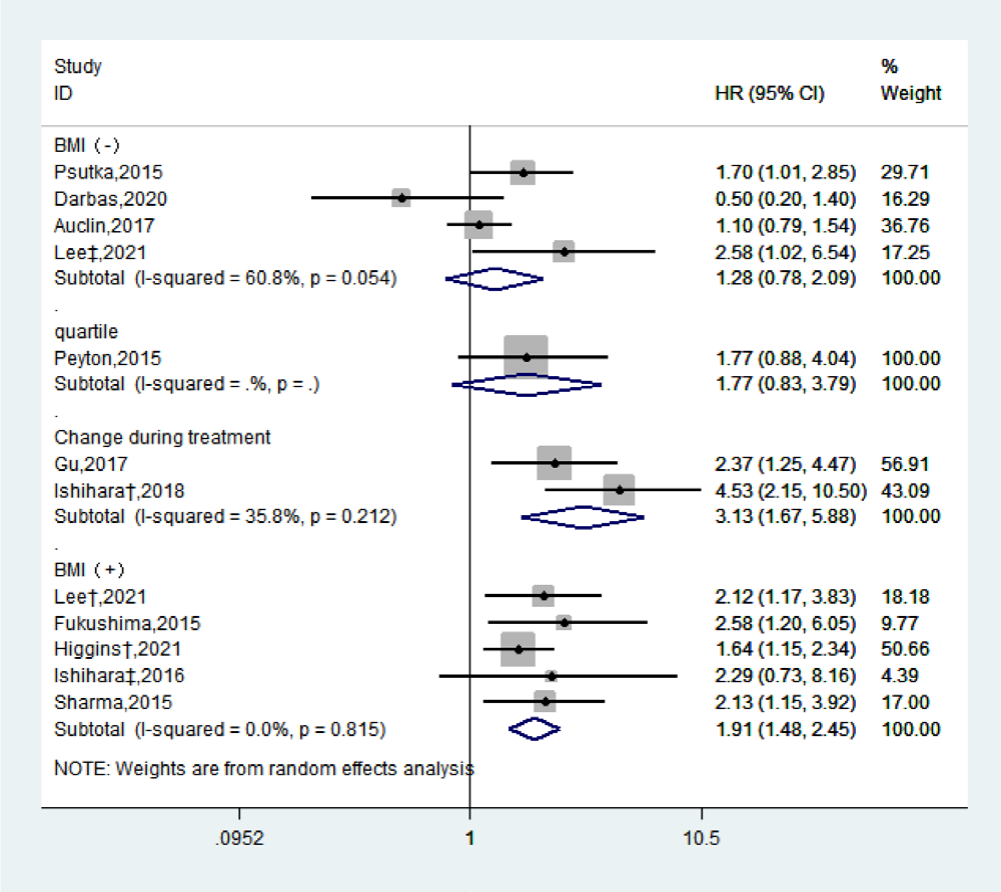
Forest map of different sarcopenia measurement indexes. BMI = body mass index, CI = confidence interval, HR = hazard ratio.

Although the vast majority of researchers have adopted the third lumbar level related to systemic skeletal muscle reserve defined by Pardo,^[27]^ which reduces heterogeneity to some extent, the actual measurement methods are quite different. Among them, 6 studies used clear gender cutoff values, and 5 studies added fat parameter (defined using BMI) to skeletal muscle indexes to help define sarcopenia, although they are not exactly the same. Noguchi^[12]^ and other scholars used the quartile of similar trend indicators, while Gu^[14]^ and Ishihara^[9]^ longitudinally observed the changes of skeletal muscle in patients with RCC during targeted treatment. We performed subgroup analysis of these four types of sarcopenia indicators respectively. The results showed that sarcopenia obesity (SMI + BMI) performed better in prognosis than the simple gender cutoff with large heterogeneity (HR: 1.91, 95% CI = [1.48, 2.45], *I*^2^ = 0%). The amount of skeletal muscle changes during treatment (HR: 3.13, 95% CI = [1.67, 5.88], *I*^2^ = 35.8%) was significantly correlated with the patient’s death outcome (Fig. 2).

### 3.3. Long-term effect process of sarcopenia

**Figure 3.**
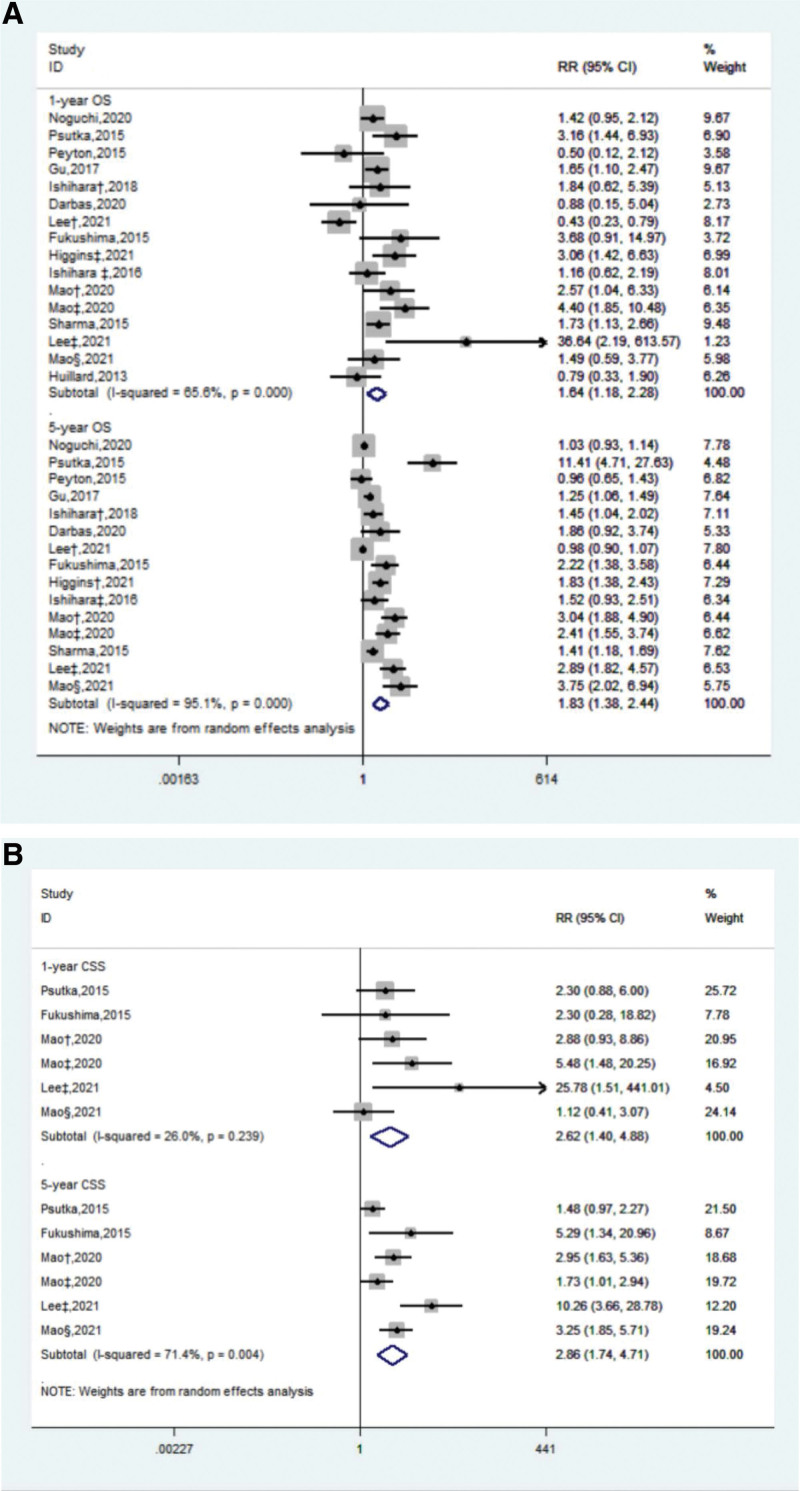
Forest map of 1, 5-year (A) all-cause mortality and 1, 5-year (B) cancer specific mortality. ^†, ‡, §^ Represent different studies published by the same author at different times. CI = confidence interval, CSS = cancer specific survival, OS = overall survival.

15 studies conducted long-term and detailed follow-up of patients. We roughly estimated the survival analysis data and conducted a summary analysis of risk. The results showed that the threat of sarcopenia to all-cause mortality existed for a long time and seemed to be aggravated (HR: 1.64, *P* < .01 to HR: 1.83, *P* < .001). This was also reflected in the risk of cancer-specific mortality, although the change was not significant (HR: 2.62, *P* < .001 to HR: 2.86, *P* < .001) (Fig. 3).

### 3.4. Meta analysis results evaluation

**Figure 4.**
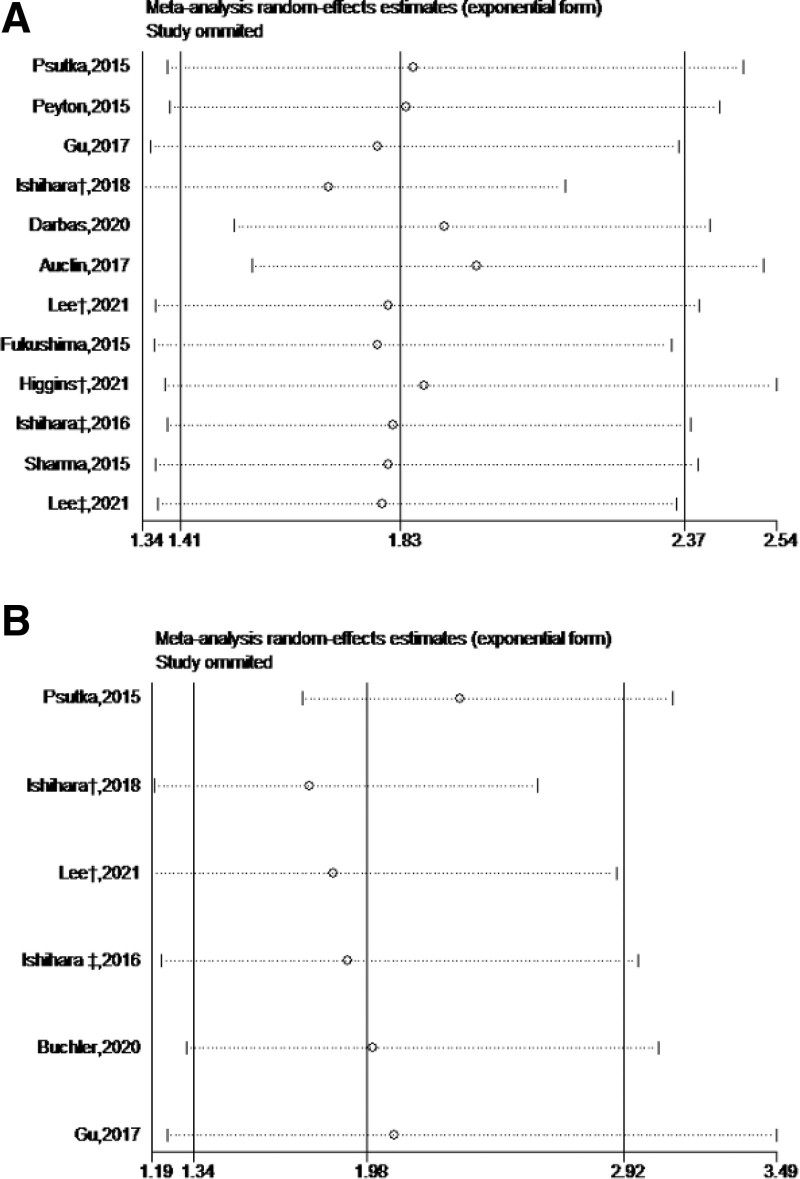
Sensitivity analysis of overall survival (A) and progression free survival (B).

**Figure 5.**
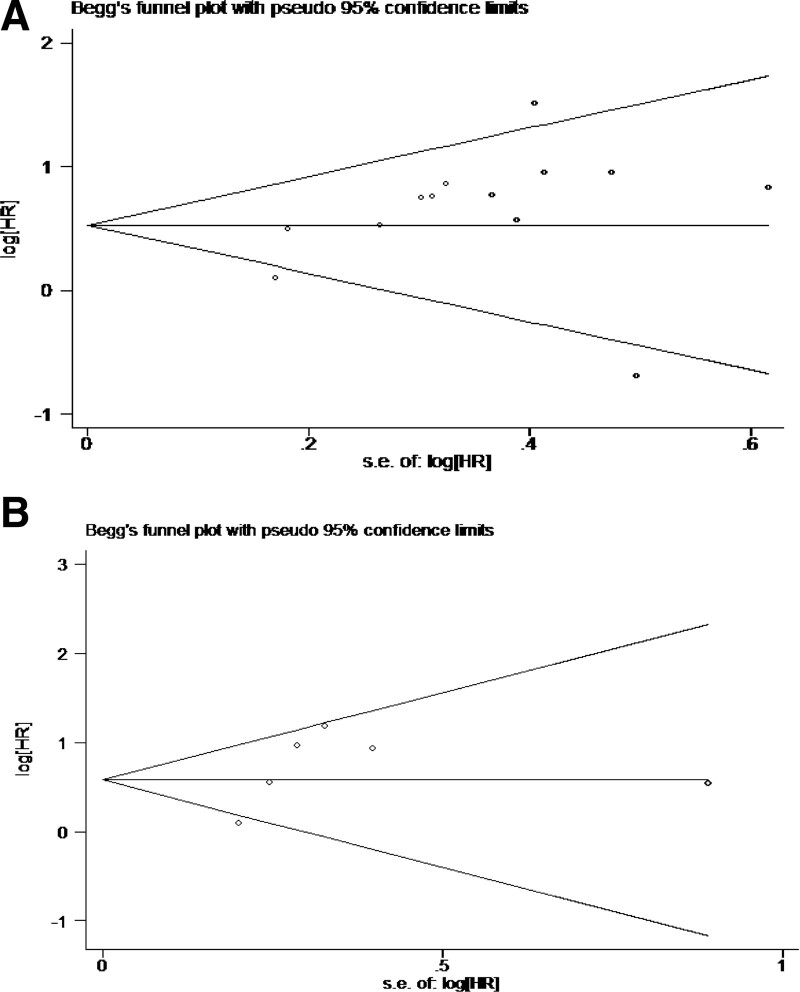
Funnel plot of overall survival (A), Begg’s test = 0.244, and progression free survival (B), Begg’s test = 0.452. HR = hazard ratio.

Although we did not limit the type of study, all included studies used a retrospective design. Most studies performed well in the review of the three quality assessment areas, and the risk of bias in some studies with relatively low quality^[13,21,26]^ is mainly due to the lack of control of the most important confounding factors such as age and the inadequacy of follow-up investigation. We conducted sensitivity analysis by deleting each study in turn, especially those with deviation risk after quality assessment (Fig. 4). Finally, the analysis results of OS showed that Auclin’s conclusion was significantly different from other studies. After analyzing the variable factors in detail, we found that it did not report the data differences between groups and the number of control variables in detail, and the study used SMI as a continuous variable instead of a binary variable. After that, we use the funnel chart to check whether there is a bias towards the research results in this field. The Begg test was used to analyze the analysis results of OS (*P* = .244) and PFS (*P* = .452). The image appearance was basically symmetrical, so there was no publication bias (Fig. 5).

## 4. Discussion

The potential interactions between sarcopenia and various diseases have been extensively studied since the WHO included sarcopenia in the ICD-10-CM (M62.84) disease list. Half of the studies we included were from 2018, obviously the relationship between changes in body composition and RCC is an emerging topic. Combined with the newly published evidence, our study confirmed the following conclusions: sarcopenia is significantly related to OS, CSS, and PFS in RCC patients. The prognostic value of this low skeletal muscle quality is independent of some common prognostic factors, such as age, tumor location and stage. In addition, the study also found that during the long-term follow-up, the effect of sarcopenia on all-cause mortality and cancer-specific mortality of RCC persisted and tended to aggravate, but the conclusion was heterogeneous. Although most studies reported the loss of follow-up in detail, almost all the imaging and laboratory data of patients came from the examination during hospitalization, and similar data were not supplemented during follow-up. Therefore, we cannot determine whether the gradual impact on the slow survival outcome came from tumor or sarcopenia. All included studies are retrospective analysis, and prospective studies controlling potential variables are needed to determine this in the future.

However, the mechanism of this specific induced death is not clear. The most common hypothesis comes from the interaction between cytokines. As an endocrine organ, skeletal muscle secretes a variety of cytokines and chemokines, which are widely involved in the systemic inflammatory state and nutritional disorder of cancer patients, and these cytokines are also important indicators of RCC specific death and progression.^[28,29]^ Meanwhile, some inflammatory factors (Interleukin-6 and tumor necrosis factor-α) can also be used as important inflammatory parameters of sarcopenia, which can also lead to anorexia by improperly regulating muscle cell metabolism (directly or indirectly leading to increased myostatin levels, up regulating ubiquitin proteasome and renin angiotensin aldosterone systems to promote protein degradation and damage skeletal muscle regeneration) and acting as an intermediary of anorexia, which may reflect potential therapeutic targets for improving the direction of muscle metabolism and realizing certain anti-tumor mechanisms.^[30,31]^

The study also found that in the context of sarcopenia, RCC patients from Asia face almost twice the risk of death in the western population, and the impact of tumor progression seems to be only for the Asian population. We speculate that this difference may come from genetic racial characteristics. On the surface, Asians have higher contents of visceral fat and subcutaneous fat. In contrast, Westerners have 15% higher skeletal muscle mass,^[32]^ which means that Asians have relatively insufficient body reserves in the face of disease risk. Based on this understanding, we found that many Eastern studies used western diagnostic criteria, and this application that does not meet the ethnic demographic characteristics may underestimate the impact of sarcopenia on Asian RCC patients. The most important thing is that there is no large sample sarcopenia study for tumor patients in Asia.

It is worth noting that in the clinical practice of treating RCC, some inflammatory markers have preliminarily proved their effectiveness in molecular targeted therapy or immunotherapy and the prediction of patient survival results, including routine C reactive protein, Neutrophil-to-lymphocyte ratio (NLR) and creatinine, but it is undeniable that such biomarkers are susceptible to immediate effects of infectious diseases and treatment modalities (such as drug-induced myelosuppression).^[33]^ In contrast, the skeletal muscle index of sarcopenia is more objective and has good repeatability. More importantly, the measurement of lumbar spine level is within the imaging scope of RCC patients, so patients do not have to have excess economic burden and trauma. Recently, the changes of skeletal muscle during treatment have gradually attracted scholars’ attention, because it is considered that it can not only judge the adverse prognosis caused by treatment methods, but also eliminate the impact of treatment measures on the accuracy of indicators, and timely correct the reserves of body composition of patients.

The definition of sarcopenia is gradually expanding, but it is still controversial. Generally speaking, the increase of skeletal muscle mass will lead to higher BMI, but it has been proved that many patients with higher BMI also suffer from sarcopenia. Therefore, some scholars adjust the measurement of skeletal muscle from fat composition. Mintziras^[34,35]^ believes that this sarcopenia obesity (SO) reflects a worse physical condition than sarcopenia. With the continuous increase of obesity rate, this adjustment may be more accurate and appropriate, which is consistent with our results. Moreover, SO seems to be able to make up for the lack of short-term prognosis of sarcopenia.^[36]^ During literature screening, we found that some prognostic studies derived from fat component indicators such as visual advertisement tissue index (VATI),^[37]^ visual fat area/subcutaneous fat area (VFA/SFA)^[38]^ have prognostic value and reflect the degree of fat infiltration in muscle, but these indicators have no reliable correlation with the sum of similar components in human body. And according to the consensus of an international expert group,^[39]^ the loss of skeletal muscle is not necessarily accompanied by significant fat changes, so this analysis does not include such related studies. However, it is worth noting that the diagnostic indicators of sarcopenia adjusted by anthropometric BMI or fat index quantified by imaging technology have unique clinical value in some diseases, but this value is only reflected when the two adjusted indicators meet the characteristics of the study cohort. Compared with SMI, the definitions of BMI and fat index are more vague, and the change direction of adjustment index seems to have different significance.^[40,41]^ For example, the increase of body mass index, it is difficult to distinguish whether this change comes from the increase of muscle mass, intractable ascites caused by tumor or fat accumulation. In addition, different definitions of sarcopenia have also led to confusion in the medical community. Some studies believe that sarcopenia can be used as an alternative indicator of cachexia, but in fact, they are only partially similar in the changes of body composition and can not be simply classified into one category. Therefore, a large sample control analysis of sarcopenia should be carried out in the future to promote the comparison between relevant studies. Because the management of cancer currently focuses on exploring new treatment objectives of personalized medicine, the physical ability of patients to receive treatment is an important factor to be considered, and its optimization may greatly improve the prognosis of patients, which can be reflected in the quality of skeletal muscle. Therefore, it is necessary to be cautious in the application of adjustment indicators, especially it is unclear which method is most beneficial to patients.

In addition, although our criteria did not exclude sarcopenia studies from the functional definition, no relevant studies were retrieved. It is worth noting that recent evidence shows that the decline pathways of skeletal muscle mass and function do not completely overlap, which means that muscle strength does not only come from mass, but may more reflect neural functional integrity or state than body composition.^[42]^ However, we also note that although muscle strength can also be used as a predictor of adverse consequences, unlike CT objective and quantitative data, functional testing (such as maximum grip strength test, 6min walking test and chair standing test) is very vulnerable to the influence of doctor experience and the current psychological and physical state of patients.^[43–45]^ Therefore, a more accurate expert consensus is needed for patients with limited physical activity such as cancer.

There are some limitations to this study. Firstly, according to the systematic review, compared with the complex and diverse sarcopenia parameters of patients with lung cancer and brain tumor, RCC patients are almost the same in measurement level and range, but we note that there are still differences in the actual cutoff value and its use in various studies. Secondly, the prognosis of RCC patients may also be affected by factors such as the degree of treatment, treatment time and hospital stay, but not all studies have reported and evaluated these factors in detail, and this cohort characteristic deviation may affect the prognosis of sarcopenia. Therefore, standardized population, consistent diagnostic thresholds and strict variable control methods are needed to transform the advantages of muscle composition. Finally, the studies we finally included are retrospective, which may lead to a certain risk of deviation, but the overall quality of the study is good.

## 5. Conclusion

The important independent prognostic value of OS, CSS and PFS in sarcopenia reflects its potential value as a routine clinical examination of RCC, but these indicators need to be strictly verified and reach the consistency of diagnostic thresholds in specific fields in the future.

## Author contributions

LYX contributed to the actual design of the research, the definition of search formulas, the acquisition of relevant data, and the writing of the original article. LJC is responsible for the review of the included studies and extracted data and final results. LWY contributed half of the data analysis and explanation, and finally determined the outline of the article. All Authors have approved the final version.

**Conceptualization:** Li Yuxuan, Liu Wenya.

**Investigation:** Li Yuxuan, Li Junchao.

**Methodology:** Li Yuxuan, Li Junchao.

**Writing – original draft:** Li Yuxuan.

**Writing – review & editing:** Liu Wenya.

## Supplementary Material


