# Supplementary figures and images for: The role of sarcopenia in treatment-related outcomes in patients with renal cell carcinoma: A systematic review and meta-analysis
Source: Medicine (Baltimore). 2022 Oct 28;101(43):e31332. doi: 10.1097/MD.0000000000031332 (PMC9622586; doi:10.1097/MD.0000000000031332)

**Supplementary Fig 1.** forest map of cancer specific survival.

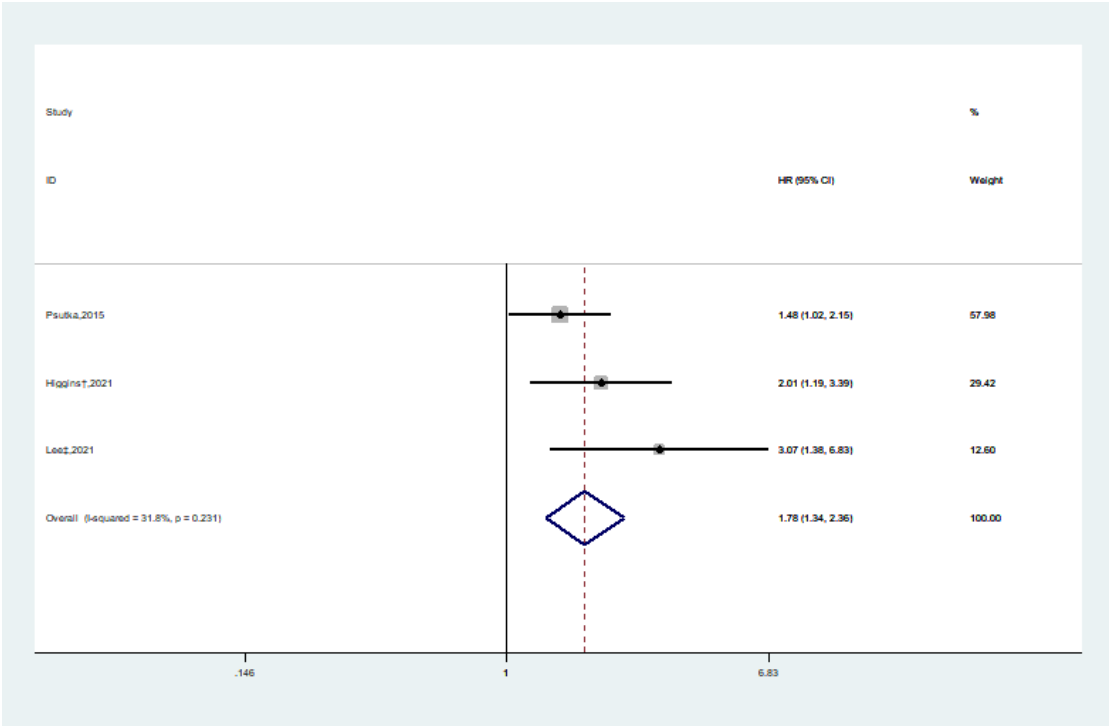

Supplement: Supplementary file 1 [file medi-101-e31332-s001.pdf]
